# Influence of Thermal Environment on Attendance and Adaptive Behaviors in Outdoor Spaces: A Study in a Cold-Climate University Campus

**DOI:** 10.3390/ijerph18116139

**Published:** 2021-06-07

**Authors:** Jiao Xue, Wei Liu, Kuixing Liu

**Affiliations:** 1 School of Design, Shanghai Jiao Tong University, Shanghai 200240, China; xuejiao@sjtu.edu.cn; 2 Division of Sustainable Buildings, Department of Civil and Architectural Engineering, KTH Royal Institute of Technology, Brinellvägen 23, 100 44 Stockholm, Sweden; wei.liu@byv.kth.se; 3 School of Architecture, Tianjin University, Tianjin 300072, China

**Keywords:** outdoor thermal comfort, occupant behavior, outdoor thermal environment, microclimate, questionnaire survey

## Abstract

Creating a favorable thermal environment in an outdoor space is essential for attracting more occupants to outdoor areas and vitalizing a city. It is possible to study occupants’ needs in an outdoor thermal environment by observing their attendance and behaviors, since people may exhibit certain adaptive measures, such as seeking shade, using parasols, etc., “vote with their feet”, or even leave the space, if they feel uncomfortable. In order to investigate the influence of thermal environment on attendance and adaptive behaviors in outdoor spaces, in this study we carried out field campaigns in a university campus in a cold-climate city. The thermal environment was monitored, while surveys of thermal perceptions and observations of attendance and adaptive behaviors were conducted. Through the data analyses, it was found that the thermal environment had a great impact on the attendance of optional activities, but necessary activities were not influenced. The greatest influence on attendance came from air temperature. The influences of wind and humidity on attendance were found to be coupled with that of air temperature. Adaptive behaviors, such as seeking shade, using parasols, changing clothes, and changing the lengths of stay, were also greatly influenced by air temperature.

## 1. Introduction

It is predicted that around two-thirds of the world’s population will live in cities [1] by 2050. Outdoor spaces provide citizens with plenty of room for their daily activities, such as exercise, resting, socializing, and attending to children [2]. High-quality spaces are needed in order to promote activity outdoors to vitalize cities [3,4]. In addition, vitalizing outdoor space can lead to energy savings in buildings [5,6,7]. Occupant behavior is an important influencer of energy and the environment [8]. According to Gehl [9], outdoor activity can be classified into three types: necessary activities, optional activities, and social activities. Gehl claimed that necessary activities, such as walking to work or school, or walking a dog, happen regardless of the thermal environment because people have no choice, but the occurrence of optional and social activities relies on a good “physical environment”. Many studies have investigated the relationship between activity level and thermal environment, but they did not distinguish among activities. 

In previous studies, the activity level was often measured by the number of people in outdoor spaces [10]. Urban climate [11] is an important driver of occupant activities in open spaces. Different studies have found contradictions regarding the exact relationship between the number of people and the thermal environment. For example, Nikolopoulou et al. [12] found that the number of people in four public open spaces in Cambridge, U.K., increased with global temperature. In Göteborg, Sweden [13,14], it was also found that more people were associated with higher mean radiant temperature. However, some studies from other regions have identified a polynomial relationship between the number of people and the thermal environment. In Guangzhou [15], Chengdu [16], and Taichung [17], as the climate became warmer, more people were present outdoors until a “cut-off” threshold. After the threshold, the number of people started to decline with increased air temperature. It may be noticed that Cambridge and Göteborg are in cold or temperate regions, while the climates in Guangzhou, Chengdu, and Taichung are warmer than those in Cambridge and Göteborg. The two sets of cities are also from different cultures: Western and Eastern Asian cultures. It is interesting to conduct a study of people in a cold-climate region in an Eastern-culture city to examine the trend between activity level and outdoor thermal environment. 

The outdoor thermal environment is constituted of four parameters: the air temperature, thermal radiation, wind speed, and humidity [18]. Previous studies explored the importance of different parameters and their influences on outdoor attendance. It was usually found that air temperature and thermal radiation had a stronger association with attendance than did wind speed and humidity [19,20]. However, the influence of these thermal environmental parameters is coupled. For example, the same level of wind speed may have different effects under different ranges of air temperature [21]. In addition, the effect of air temperature may change under different levels of humidity. These coupled influences from these outdoor thermal environment parameters were not explored in previous studies. 

In an outdoor space, people may exhibit many behaviors in order to better adapt themselves to the thermal environment. These behaviors include seeking shade, using shading devices, changing clothes, changing their time of stay, etc. A number of researchers [17,22,23,24,25] have directly asked respondents to select the actions they would take to adapt to the outdoor environment. Among all the choices, seeking shade was the most frequently selected behavior. Most previous investigations only asked subjects to choose from several adaptive behaviors [23,24,25]. The observations and correlation analyses were usually performed for a single behavior, such as seeking shade or changing clothes, while no comprehensive studies concerning adaptive behavior with regard to the outdoor thermal environment have been conducted. 

In order to study the influence of various thermal environment parameters on outdoor attendance in a cold-climate region, and to further quantify their impact on adaptive behaviors, we carried out a half-year study concerning attendance and adaptive behaviors in outdoor open spaces on a university campus in a cold-climate city in China. The emphasis of the current study is on the impact of thermal environment parameters on attendance and adaptive behaviors.

## 2. Methods

Field campaigns were conducted on a university campus in Fuxin, China, to collect data on the outdoor thermal environment, occupant perceptions, and corresponding attendance and adaptive behaviors. This section firstly describes the studied sites and presents the climate of Fuxin. Then, the measurement campaign is described in detail. 

### 2.1. Site Description

**Figure 1:**
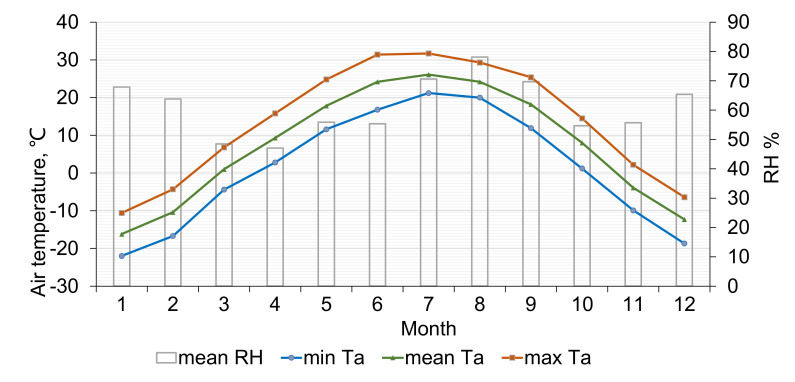
Monthly mean/maximum/minimum air temperature and mean relative humidity in Fuxin from 1971 to 2003. Source: Meteorological database for thermal environment analysis of Chinese buildings.

Our study was conducted in Fuxin, a city in Liaoning Province, located in the northeastern part of China. Based on the Koppen Climate Classification [26], Fuxin has a monsoon-influenced, humid continental climate (Dwa), characterized by long cold winters and short and mild summers. Figure 1 shows the average, maximum, and minimum air temperature (*T_a_*) and mean relative humidity (*RH*) values in Fuxin between 1971 and 2003. The average *T_a_* is highest in July at 26.1 °C and lowest in January at −16.2 °C. The maximum *T_a_* is highest in June at 31.4 °C and lowest in January at −22.0 °C. The relative humidity is high in winter and summer, around 70%, and low in spring and autumn. 

**Figure 2:**
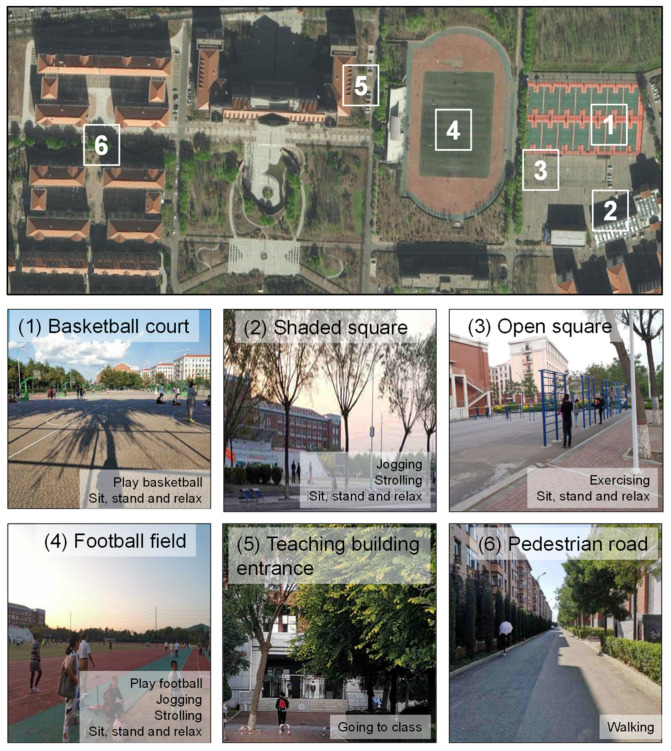
Selected places of study and corresponding activities at each place: (**1**) Basketball court, (**2**) Shaded square, (**3**) Open square, (**4**) Football field, (**5**) Teaching building entrance, and (**6**) Pedestrian road.

The field campaign of outdoor activities was carried out on the campus of Liaoning Technical University. The campus has a wide variety of thermal environments for different outdoor activities. Figure 2 shows the studied outdoor spaces, including basketball courts (Place #1), a shaded square (Place #2), an open square (Place #3), a football field (Place #4), the entrance of one teaching building (Place #5), and a pedestrian road (Place #6). The main users of the campus were university students. Optional and social activities, such as playing football and basketball, jogging, strolling, or just standing, relaxing and chatting happened at Place #1 to Place #4. Place #5, the entrance of a teaching building, was selected to study the influence of the thermal environment on going to classrooms, which is considered a necessary activity. Place #6 is a pedestrian road with partial shading for the study of shade-seeking behavior.

### 2.2. Measurement Campaign

In order to gather data for the analysis of the influence of the thermal environment on space attendance and adaptive behavior in the outdoor spaces, 12 field campaigns were conducted from 13 June 2019 to 15 December 2019 on sunny days, from 8:00 a.m. to 18:00 p.m. on summer days and from 8:00 a.m. to 17:00 p.m. on autumn and winter days. To avoid the influence of holidays, all campaigns were performed on workdays. The field campaigns included three parts: thermal environment monitoring, questionnaire survey, and activity observation. 

**Figure 3:**
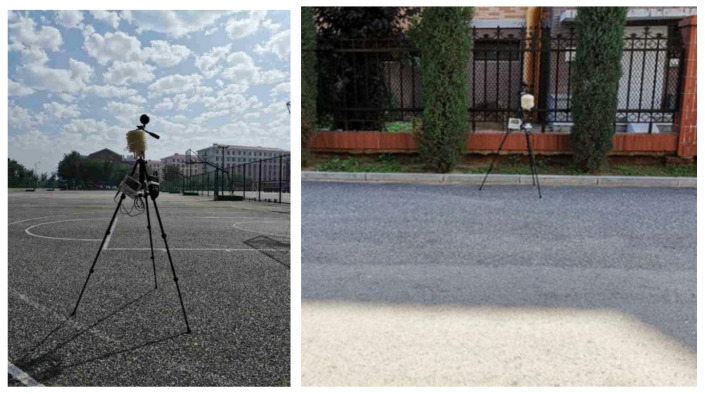
Measurement of thermal environment parameters under the sun (**left**) and under shade (**right**).

The air temperature (*T_a_*), relative humidity (*RH*), wind speed (*WS*), and globe temperature (*T_g_*) were measured by sensors mounted at a 1.5 m height on a tripod, as shown in Figure 3. The specifications of the sensors are shown in Table 1. The parameters were manually recorded every 10 min. Since the thermal environments in sunny and shaded spaces may be different, especially regarding the globe temperature, two sets of instruments were used to record the respective thermal environments in the sunny and shaded spaces. 

The globe temperature was used to derive the mean radiant temperature (*T_mrt_*) based on the following equation from the ISO 7726 standard [27]:

(1) $$T_{mrt}={\left[{\left(T_{g}+273\right)}^{4}+\frac{1.10\times 10^{8}WS^{0.6}}{\varepsilon D^{0.4}}\left(T_{g}-T_{a}\right)\right]}^{\frac{1}{4}}-273$$

where *D* is the globe diameter (0.07 m in this study) and its emissivity (0.95 for a black globe), *T_g_* is the globe temperature (°C), and *WS* is wind speed (m/s). *T_mrt_* is the uniform surrounding temperature in an imaginary enclosure in which the radiant heat transfer from a human body to the enclosure surfaces is equal to the heat transfer to the surfaces of an actual enclosure with non-uniform temperatures. The difference between *T_mrt_* and *T_a_* can be used to indicate the intensity of outdoor thermal radiation. For example, in shaded places, *T_mrt_* and *T_a_* are approximately equal. In sunny places, depending on the intensity of the solar radiation, *T_mrt_* may be much greater than *T_a_*. 

Questionnaire surveys were distributed at Places #1, #2, #3, and #4 in order to obtain the subjective feelings of occupants in the spaces. To reduce the rejection rate, the questionnaire was designed to be easily completed within one minute. The first part of the questionnaire included questions concerning personal info such as gender, age, and clothing. The second part of the questionnaire was mainly about people’s perceptions of thermal comfort, thermal satisfaction, thermal preference, and thermal sensation. One very important question was about people’s thermal sensation, which was rated using the ASHRAE [28] seven-point scale (−3 = cold; −2 = cool; −1 = slightly cool; 0 = neutral; 1 = slightly warm; 2 = warm; 3 = hot). A total of 664 questionnaires were collected.

While monitoring the thermal environment and administrating surveys, observations of the number of people and their adaptive behaviors were carried out simultaneously. The number of people performing optional activities such as playing football, basketball, resting, jogging, and chatting was recorded every 20 min at Place #1 to Place #4. The gender of participants and specific activity were also recorded. In Place #2, the times that occupants entered and left that space were documented to calculate the length of stay. In Place #5, the number of students attending class was recorded to study the impact of the thermal environment on necessary activity. In Place #6, the percentages of people choosing to walk under the shade or using parasols were studied. 

### 2.3. Statistical Analysis Method

To investigate which thermal environment parameter has a greater association with and impact on occupant attendance in the outdoor spaces, Pearson correlation coefficients and standardized regression coefficients were calculated. We firstly used the Pearson correlation coefficient, which is a value between −1 and 1, to measure the strength and direction of associations between occupant attendance and the thermal environment parameters (*T_a_*, *RH*, *WS*, and *T_mrt_* − *T_a_*). Second, the standardized regression coefficients from the multiple linear regression between the attendance (dependent variable) and thermal environment parameters (independent variables) were used to compare the contributions from different thermal environment parameters to variations in occupant attendance. The weights of different variables were determined by dividing the absolute standard regression coefficient by the sum of all the absolute values of standard regression coefficients. The statistical evaluations were conducted using the IBM SPSS Statistics 23 program. 

## 3. Results

Using the collected data concerning the outdoor thermal environment, outdoor space attendance, and adaptive behaviors, this section presents the influence of the thermal environment on necessary and optional behaviors, along with adaptive behaviors such as seeking shade, using parasols, changing clothing insulation levels, and changing the length of stay. 

### 3.1. Thermal Sensation Distribution

**Figure 4:**
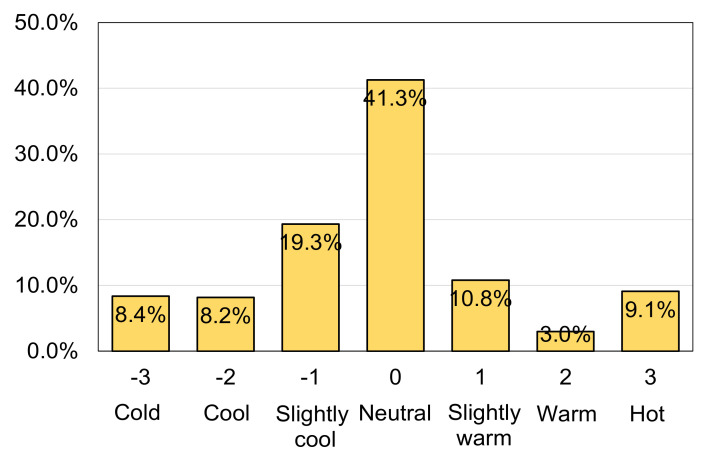
Distribution of the thermal sensation votes (TSVs) obtained during this study.

Figure 4 shows the distribution of thermal sensation votes during the field survey. The distribution demonstrates that the study covered a wide range of thermal environments. The most frequently perceived sensation was “neutral”, with 41.3% of votes. The central three sensations accounted for over 70% of votes, showing a high overall satisfaction rate. 

### 3.2. Occupant Attendance

Necessary and Optional Activities

**Figure 5:**
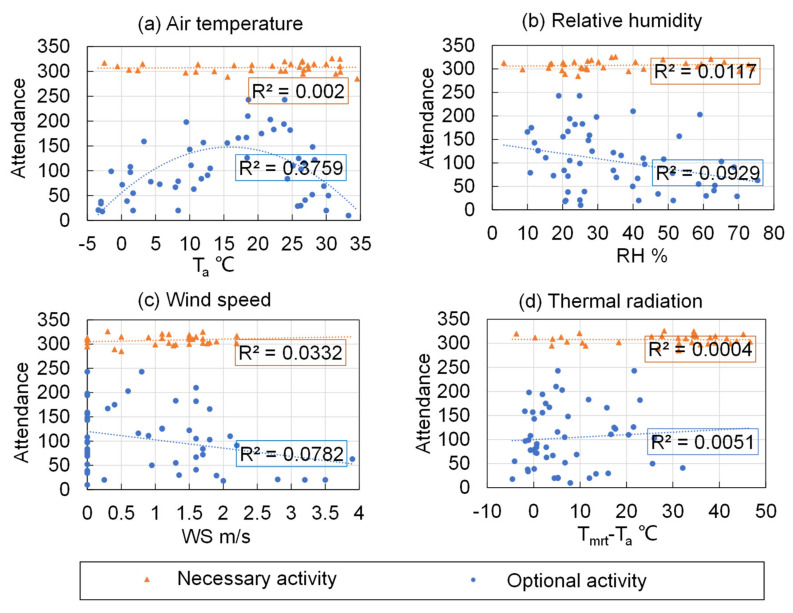
The relationships between different thermal environment parameters and attendance of necessary and optional activities for: (**a**) Air temperature, (**b**) Relative humidity, (**c**) Wind speed, and (**d**) Thermal radiation.

Figure 5 compares the relations between the monitored thermal environment parameters (air temperature *T_a_*, relative humidity *RH*, wind speed *WS*, and the difference between the mean radiant temperature and air temperature *T_mrt_* − *T_a_*) for optional and necessary activities. It can be seen that the attendance at necessary activities was constantly around 300 persons and was not influenced by the four thermal environment parameters at all, since students have to go to class regardless of the thermal environment. By comparison, the occurrence at optional activities varied greatly. 

Figure 5 further shows the influence of different thermal environment parameters on the number of people engaged in optional activities. Air temperature demonstrated a polynomial relationship with the number of people in attendance. The number of people increased until the air temperature reached 20 °C. When the air temperature was greater than 20 °C, the attendance started to decline. The other three parameters had much weaker associations with attendance. 

To further quantify the associations, the data were separated by *T_a_* of 20 °C, and Pearson’s correlation coefficients were calculated between the attendance and the four thermal environment parameters for air temperatures above and below 20 °C. Table 2 shows the calculated Pearson’s correlations coefficients along with their *p*-values. Air temperature had the greatest absolute correlation with attendance among all the investigated parameters. It was also found that when *T_a_* < 20 °C, wind speed had a moderate negative association with attendance, showing that the number of people decreased with increasing wind speed. When *T_a_* was greater than 20 °C, relative humidity had a moderate negative relationship with attendance, indicating fewer people under higher *RH*. It was found that *WS* when *T_a_* > 20 °C, *RH* when *T_a_* < 20 °C, and *T_mrt_* − *T_a_* each had a low degree of correlation with attendance. 

**Figure 6:**
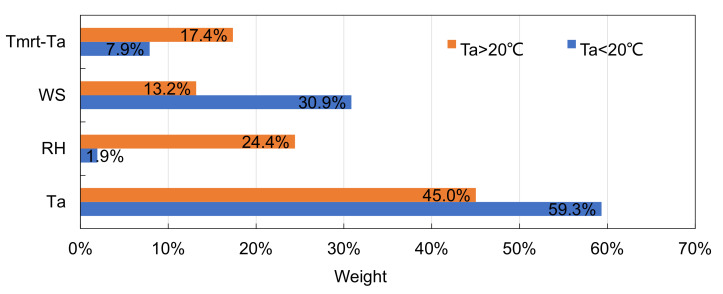
Weights of different thermal environment parameters on attendance of optional activities when air temperature was above or below 20 °C.

The relative weights for different thermal environment parameters on attendance for *T_a_* < 20 °C and *T_a_* > 20 °C were determined from the standard regression coefficients, and these are shown in Figure 6. When *T_a_* < 20 °C, air temperature contributed around 60% to attendance, followed by wind speed, from which the contribution was around 30%. When *T_a_* > 20 °C, the contribution from air temperature was as large as 45%, and relative humidity and thermal radiation showed contributions of 24% and 17%, respectively.

**Figure 7:**
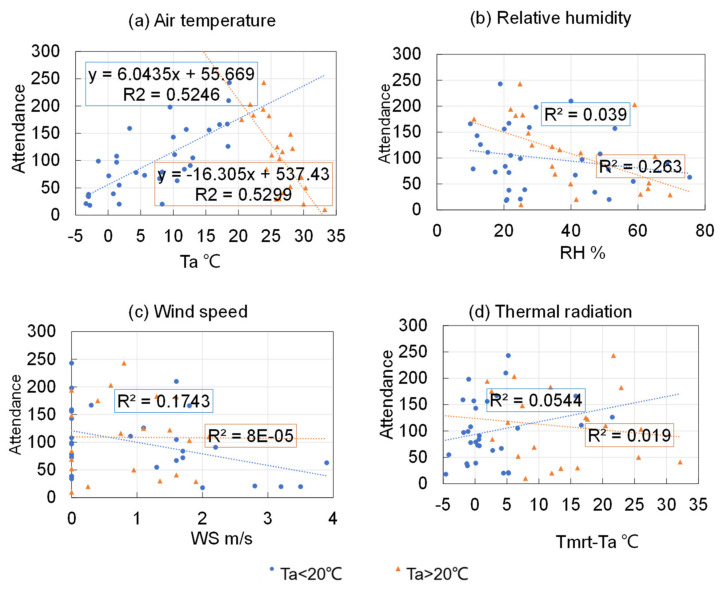
Occupant attendance of optional activities under different air temperatures for: (**a**) Air temperature, (**b**) Relative humidity, (**c**) Wind speed, and (**d**) Thermal radiation.

To further analyze the sensitivity of the number of people to the thermal environment in “cold” and “hot” seasons, the data set was split at *T_a_* = 20 °C, and the number of people was plotted with respect to *T_a_*, *RH*, *WS*, and *T_mrt_* − *T_a_* for *T_a_* < 20 °C and *T_a_* > 20 °C in Figure 7. Figure 7a shows that the occupants were more sensitive to hot than to cold. When *T_a_* < 20 °C, every 1 K decrease in air temperature resulted in a reduction of 6 occupants, while for *T_a_* > 20 °C, 16 people left the outdoor areas for every 1 K increase in air temperature. 

**Figure 8:**
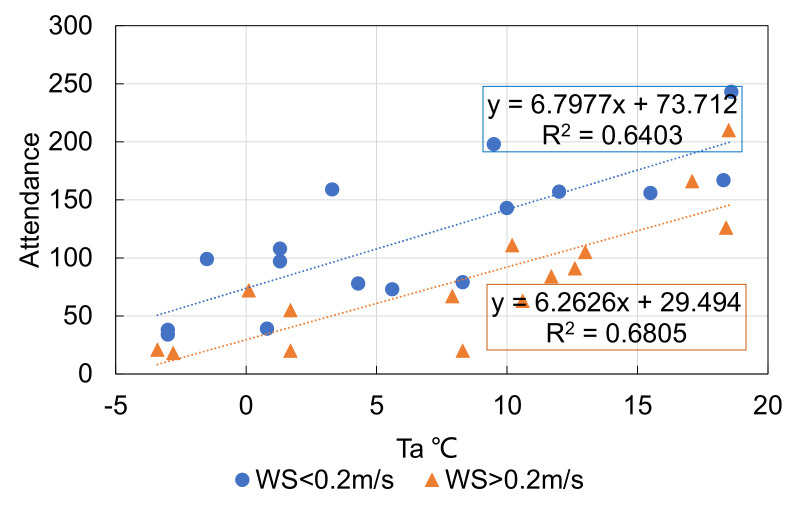
Wind speed’s influence on attendance of optional activities when *T_a_* < 20 °C.

**Figure 9:**
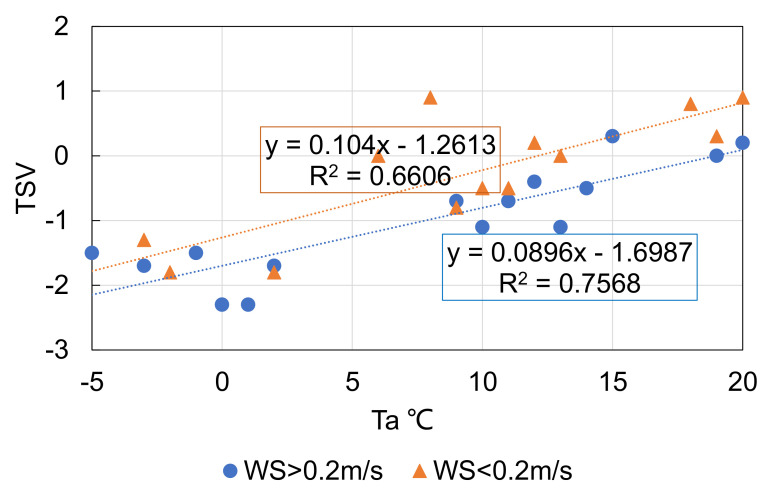
Wind speed’s influence on TSV when *T_a_* < 20 °C.

The previous analyses showed different associations between thermal environment and attendance when *T_a_* was above or below 20 °C. The influence of wind speed on attendance when *T_a_* < 20 °C is further depicted in Figure 8, showing the number of people with respect to air temperature for wind speed higher or lower than 0.2 m/s. The sensitivities of attendance to air temperature for the two different sets of wind speed were similar, but the number of people for a wind speed of >0.2 m/s was about 50 less than when the wind speed was less than 0.2 m/s. Higher wind speed when *T_a_* < 20 °C caused a 0.5 to 1.0 lower scale category of thermal sensation, as shown in Figure 9. As a result, people were less willing to engage in optional activities. 

**Figure 10:**
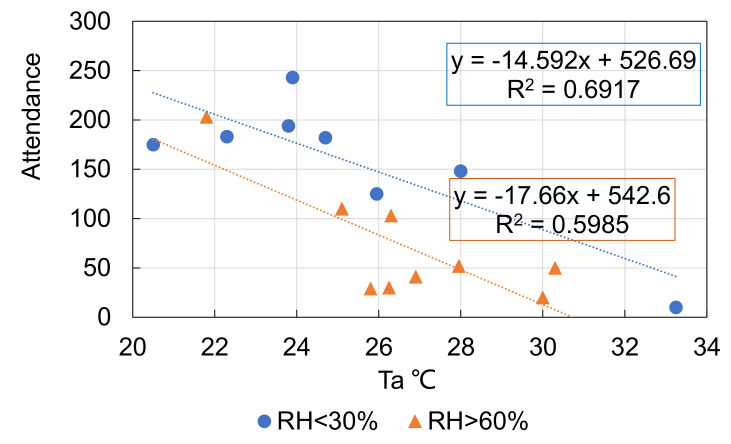
*RH* level’s influence on the attendance of optional activities when *T_a_* > 20 °C.

Similar to the analysis of the influence of wind speed in a cold season in Figure 8, Figure 10 shows the attendance of optional activities when *T_a_* was greater than 20 °C for *RH* < 60% and *RH* > 60%. Lower attendance was found for higher levels of *RH*. A high level of relative humidity under a high air temperature is unfavorable for thermal comfort. As a result, the attendance lowered under such a thermal environment. 

### 3.3. Adaptive Behaviors

**Figure 11:**
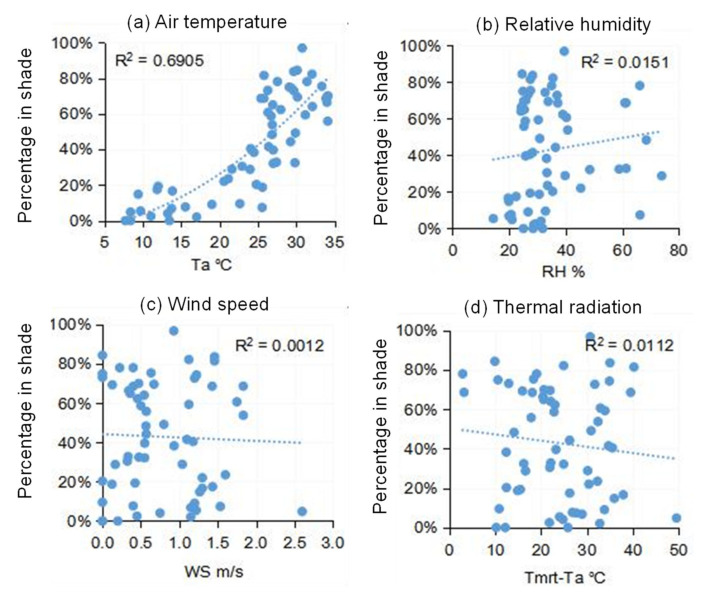
The percentage of people walking in shade with respect to *T_a_*, *RH*, *WS*, and *T_mrt_* − *T_a_* for: (**a**) Air temperature, (**b**) Relative humidity, (**c**) Wind speed, and (**d**) Thermal radiation.

People adapt themselves to the environment by exhibiting different behaviors. Seeking sun or shade is an important adaptive measure to adapt to the thermal environment. We recorded the percentage of pedestrians walking under the shade in Place #6 (a pedestrian road) and plotted that percentage with respect to the four different thermal environment parameters in Figure 11. It can be seen that the correlations between the percentage of people in shade and relative humidity, wind speed, and *T_mrt_* − *T_a_* were very low. The air temperature was the single significant parameter affecting shade-seeking behavior. When air temperature was lower than 20 °C, the percentage of people in the shade was less than 20%. The percentage of people in the shade significantly increased after 20 °C. At 27 °C air temperature, 50% of people chose to walk under the shade, and the percentage increased to 80% at an air temperature of 33 °C. 

**Figure 12:**
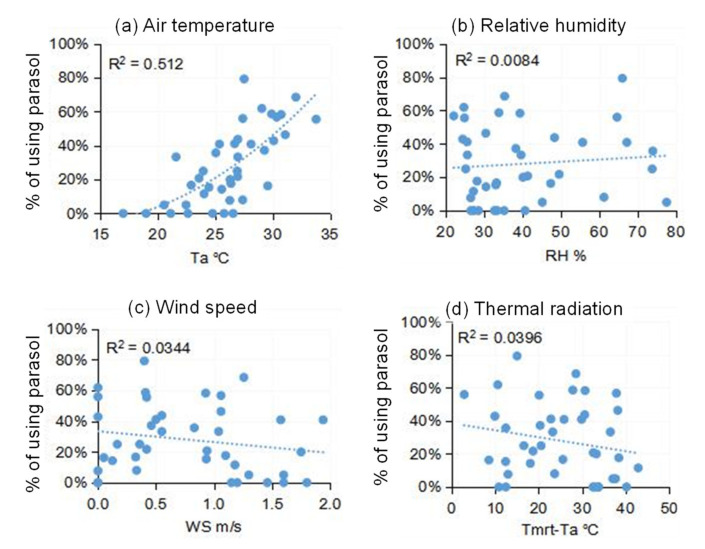
Percentage of women using parasols with respect to *T_a_*, *RH*, *WS*, and *T_mrt_* − *T_a_* for: (**a**) Air temperature, (**b**) Relative humidity, (**c**) Wind speed, and (**d**) Thermal radiation.

Using a parasol to shade oneself from the sun is another important adaptive measure in addition to seeking building or vegetation shade. According to our observations, men and women showed a distinctive trend in using parasols. Very few men used parasols during our field campaign. Thus, we only used the data of women to investigate the parasol use behavior. Figure 12 presents the associations between thermal environmental parameters and the percentage of women using a parasol. The trend in Figure 12 is similar to that in Figure 11, with air temperature playing the single most important role. The rate of parasol use as a percentage increased with air temperature. Only a few women used parasols when the air temperature was lower than 20 °C. At air temperatures of around 30 °C, the percentage increased to 50%. In Eastern Asian culture, a fair skin tone is considered an important component of beauty, so the use of parasols is a measure for women to prevent sun tanning. However, our study found that the thermal effect is a significant influence factor in the use of a parasol. In other words, women only increase the use of parasols with an increase in air temperature, even though the sun affects skin tone regardless of the air temperature. 

**Figure 13:**
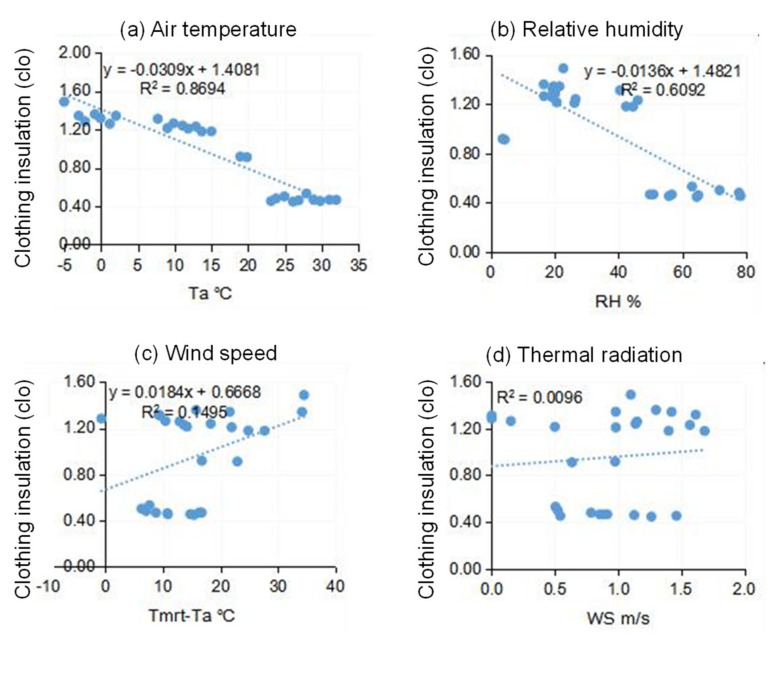
The clothing insulation level with respect to *T_a_*, *RH*, *WS*, and *T_mrt_* − *T_a_* for: (**a**) Air temperature, (**b**) Relative humidity, (**c**) Wind speed, and (**d**) Thermal radiation.

The amount of clothing worn by a person affects the heat balance of their body and has a considerable impact on thermal comfort. This study recorded the number of clothes worn from answers in questionnaires, and we plotted the clothing insulation level with respect to different thermal environment parameters in Figure 13. Air temperature was still an important driver of clothing change behavior. Subjects wore around 1.4 clo of clothes when the air temperature was around 0 °C. As the air temperature increased, subjects decreased the amount of clothes worn. The clo value approached 0.4 clo for air temperatures greater than 23 °C and remained almost constant thereafter. The relative humidity also impacted the clothing choice, as seen from Figure 13. 

**Figure 14:**
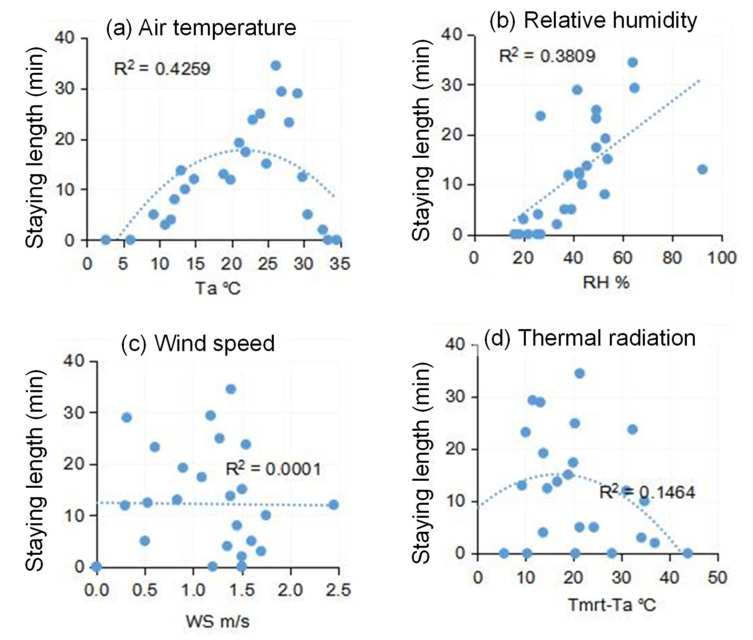
The relationships between staying length and *T_a_*, *RH*, *WS*, and *T_mrt_* − *T_a_* for: (**a**) Air temperature, (**b**) Relative humidity, (**c**) Wind speed, and (**d**) Thermal radiation.

The length of stay in spaces is also an important indicator of people’s satisfaction with the thermal environment, since people tend to stay longer if the environment is more favorable. Figure 14 plots the recorded length of stay under different thermal environment parameters. Similar to the trend for attendance, air temperature exhibited a polynomial relationship with the length of stay. Occupants stayed longer with increasing air temperature, until around 27 °C. After 27 °C, the length of stay declined with the rise in *T_a_*. When *T_a_* was <10 °C or >30 °C, the length of stay was less than 5 minutes. The increase in relative humidity also had a negative influence on people’s willingness to stay in the outdoor space. The effect of wind speed was unclear, but thermal radiation exhibited a weak polynomial correlation with the length of time stayed. 

## 4. Discussion

Studies in Cambridge, U.K. [12] and Göteborg, Sweden [13,14] found that attendance monotonically increased when the climate became warmer, while in studies conducted in Eastern Asia, including in Taiwan [17,29,30], Guangzhou [15], Chengdu [16], Wuhan [31], and Tianjin [32], polynomial relationships were found between attendance and climate. Although the studies in Cambridge, U.K. and Göteborg, Sweden were conducted in mild or cold-climate regions, a closer look at the literature revealed that the highest recorded air temperature in those studies was close to 30 °C. The current study was also conducted in a cold-climate city, and it was found that the attendance started to decrease as early as the air temperature reaching only 20 °C. As a result, cultural factors may be an important reason for the distinctive results between the studies conducted in Europe and in Eastern Asia. 

The study showed that, even in a cold-climate city such as Fuxin, people still need shade to adapt to the environment. Thus, it is suggested that a diversified thermal environment, such as with sunny and shaded sub-places, should be created in outdoor spaces to provide adaptive opportunities. 

One factor that should be noted is that the main object of this study was university students. Since university students may come from different climate zones and have distinctive thermal histories, caution should be taken in generalizing the conclusions to a wider population. Further studies should be conducted on local residents to obtain further information.

## 5. Conclusions

The current study investigated the influence of the thermal environment on attendance and adaptive behaviors in outdoor spaces on a university campus through the monitoring of the microclimate, distribution of questionnaire surveys, and observation of attendance and adaptive behaviors. It was found that the thermal environment, especially air temperature, had a significant influence on occupant attendance and adaptive behavior. As a result, it is important to consider the thermal environment in outdoor space design in order to vitalize open spaces and the city as a whole. More specifically, the findings are as follows:

- While necessary activity occurred at a constant rate regardless of the thermal environment, optional activities were significantly influenced by changes in air temperature. When the air temperature was less than 20 °C, the attendance of optional activities increased with the increase in air temperature. After 20 °C, the attendance reduced with increasing air temperature. It was also found that high air temperature had a more significant impact on attendance than did low air temperature.
- The influence of wind speed and relative humidity on attendance was coupled with air temperature. When the air temperature was <20 °C, higher wind speed decreased the number of people participating in optional activities. When the air temperature was >20 °C, higher relative humidity reduced attendance.
- Air temperature exhibited an important influence on adaptive behaviors, including seeking shade, using parasols, changing clothes, and changing the length of stay. Higher air temperature led to an increased percentage of people walking in the shade, an increased percentage of women using parasols, and decreased clothing insulation. A polynomial relationship was found between air temperature and the length of stay.

## Figures and Tables

**Table 1:** Specifications of the sensors used in the field campaign.

Parameter	Sensor	Range	Accuracy	Resolution
Air temperature	S500-EX HUATO	−40~85 °C	±0.5 °C	0.1 °C
Relative humidity	S500-EX HUATO	0~100%	±5%	0.1%
Wind speed	GM 8901	0.3~45 m/s	±3%	0.1 m/s
Globe temperature	JTR 04	10–80 °C	±0.5 °C	0.1 °C

**Table 2:** Pearson correlation coefficients for different environmental parameters and attendance.

		*T_a_*	*RH*	*WS*	*T_mrt_ − T_a_*
*T_a_* < 20 °C	Pearson correlation coefficient	0.724	−0.197	−0.417	0.233
	*p*-value	0.000	0.287	0.019	0.206
*T_a_* > 20 °C	Pearson correlation coefficient	−0.729	−0.513	−0.02	−0.138
	*p*-value	0.000	0.017	0.931	0.551

## Data Availability

Data will be available upon request.

## Nomenclature

*T_a_*	air temperature, °C
*RH*	relative humidity, %
*WS*	wind speed, m/s
*T_g_*	globe temperature, °C
*T_mrt_*	mean radiant temperature, °C
TSV	thermal sensation vote
